# P-93 - WASTEWATER BASED EPIDEMIOLOGY FOR ILLICIT DRUG SURVEILLANCE IN A SMALL ISLAND COMMUNITY: TRENDS, RISKS, AND HARM REDUCTION POTENTIAL

**DOI:** 10.1093/pubmed/fdag046.095

**Published:** 2026-07-09

**Authors:** Dr Rebecca Rowley, Ms Theresa Thornton, Dr Matt Tyrer

**Affiliations:** Public Health Isle Of Man; Public Health Isle Of Man; Public Health Isle Of Man

## Abstract

**Background:**

Small island communities experience unique challenges around illicit drug use. Geographic barriers create fluctuating supply, contributing to high levels of poly-drug use and increased overdose risk. Limited routine health data and restricted testing of seized substances further constrain understanding of population drug trends. Wastewater-based epidemiology (WBE) has emerged as a promising method to generate accurate, population-level estimates of drug consumption and to identify emerging substances of concern. However, ethical considerations and the need for strong public understanding are amplified in close-knit communities. Ensuring confidence in WBE for public health harm reduction, rather than criminalisation, and the anonymity of surveillance is essential for public and political support.

**Objectives:**

This project aims to use WBE to provide routine surveillance of illicit drugs. It will generate population-level consumption estimates and trend data, with the potential for early detection of high-risk substances, including synthetic opioids, enabling rapid and coordinated harm reduction responses.

**Methods:**

The project builds on existing wastewater sampling infrastructure from a 2022 pilot that demonstrated feasibility. Sampling will be twice weekly from a single treatment plant covering approximately 65% of the population. Target substances include cocaine, heroin, methamphetamine, amphetamine, MDMA, cannabis, ketamine, synthetic opioids, and novel psychoactive substances.

**Results:**

Population normalised consumption estimates may be achievable if accurate household usage data can be obtained; if not, WBE can still provide objective, consistent trend data. In poly-drug using populations, surveillance enables early identification of shifts toward high-risk substances and alerts for harmful drugs such as synthetic opioids. This intelligence can support timely public health action, including drug alerts and enhanced harm reduction measures such as wider Naloxone distribution.

**Conclusions:**

WBE offers a cost effective and non-intrusive approach to monitoring illicit drug trends in small island settings. When integrated into a broader harm reduction framework, WBE can meaningfully strengthen the public health response.

